# Influence of *M. tuberculosis* Lineage Variability within a Clinical Trial for Pulmonary Tuberculosis

**DOI:** 10.1371/journal.pone.0010753

**Published:** 2010-05-20

**Authors:** Payam Nahid, Erin E. Bliven, Elizabeth Y. Kim, William R. Mac Kenzie, Jason E. Stout, Lois Diem, John L. Johnson, Sebastien Gagneux, Philip C. Hopewell, Midori Kato-Maeda, the Tuberculosis Trials Consortium

**Affiliations:** 1 Division of Pulmonary and Critical Care Medicine, Francis J. Curry National Tuberculosis Center, San Francisco General Hospital, University of California San Francisco, San Francisco, California, United States of America; 2 Division of Tuberculosis Elimination, Centers for Disease Control and Prevention, Atlanta, Georgia, United States of America; 3 Duke University Medical Center, Durham, North Carolina, United States of America; 4 Case Western Reserve University, Cleveland, Ohio, United States of America; 5 Medical Research Council, National Institute for Medical Research, London, United Kingdom; 6 Swiss Tropical and Public Health Institute, Basel, Switzerland; University of Stellenbosch, South Africa

## Abstract

Recent studies suggest that *M. tuberculosis* lineage and host genetics interact to impact how active tuberculosis presents clinically. We determined the phylogenetic lineages of *M. tuberculosis* isolates from participants enrolled in the Tuberculosis Trials Consortium Study 28, conducted in Brazil, Canada, South Africa, Spain, Uganda and the United States, and secondarily explored the relationship between lineage, clinical presentation and response to treatment. Large sequence polymorphisms and single nucleotide polymorphisms were analyzed to determine lineage and sublineage of isolates. Of 306 isolates genotyped, 246 (80.4%) belonged to the Euro-American lineage, with sublineage 724 predominating at African sites (99/192, 51.5%), and the Euro-American strains other than 724 predominating at non-African sites (89/114, 78.1%). Uneven distribution of lineages across regions limited our ability to discern significant associations, nonetheless, in univariate analyses, Euro-American sublineage 724 was associated with more severe disease at baseline, and along with the East Asian lineage was associated with lower bacteriologic conversion after 8 weeks of treatment. Disease presentation and response to drug treatment varied by lineage, but these associations were no longer statistically significant after adjustment for other variables associated with week-8 culture status.

## Introduction

*Mycobacterium tuberculosis* is genetically more diverse than previously believed [1]. With the availability of whole genome sequences of several clinical isolates [2], [3], [4], the genetic diversity of *M. tuberculosis* has been chronicled in the form of insertions, deletions, and single nucleotide polymorphisms (SNP). These polymorphisms have enabled researchers to genotype and classify clinical strains of *M. tuberculosis* into different groups. Recently, a group of insertions/deletions (also known as region of difference or RD) that are unique event polymorphisms and define a robust phylogeny were described. Based on this phylogeny, six lineages of *M. tuberculosis* have been defined [5]. Studies suggest that certain lineages preferentially cause active tuberculosis amongst human populations with distinct genetic ancestry [6], [7]. Some *M. tuberculosis* strains have been associated with increased transmission and some are reported to induce stronger host inflammatory responses when compared with the laboratory strain H37Rv [8], [9], [10]. Given the diversity in insertions, deletions and SNPs seen in *M. tuberculosis*, it is plausible that the genetics of the pathogen play a role not only in the natural history of tuberculosis infection and disease [11], but also in presentation of disease and response to treatment.

In this study, we sought to determine the *M. tuberculosis* lineages of isolates from pulmonary tuberculosis patients from four continents who were enrolled in a clinical trial, USPHS Tuberculosis Trials Consortium (TBTC) Study 28, a phase 2 trial designed to compare the antimicrobial activity and safety of moxifloxacin versus isoniazid when used in combination with rifampin, pyrazinamide, and ethambutol during the first 8 weeks of therapy for smear positive, culture confirmed, drug-susceptible pulmonary tuberculosis [12]. Secondarily, we explore in a limited analysis the influence of lineage and sublineage on presentation of disease and response to treatment using the trial's standardized microbiologic endpoints.

## Materials and Methods

### Ethics Statement

The parent TBTC clinical trial, Study 28, was approved by both CDC and local institutional review boards. Written informed consent was provided by each study participant. In addition, the institutional review board at UCSF approved this ancillary study to determine the lineage of *M. tuberculosis* and to analyze its influence on presentation of disease and response to treatment.

### Study Population and Setting

TBTC Study 28 was a randomized, placebo-controlled, double-blind, phase 2 clinical trial to evaluate the antimicrobial activity and safety of moxifloxacin substituted for isoniazid during the first 2 months (intensive phase) of combination treatment for smear positive, culture-confirmed, drug-susceptible pulmonary tuberculosis. Sputum culture status on both liquid and solid media at the completion of 8 weeks of treatment was the primary endpoint and was used as a surrogate endpoint for treatment efficacy. Adults were enrolled from February 2006 through March 2007 at 26 sites, including 21 sites in the United States and one site each in Brazil, Canada and Spain (collectively categorized as non-African region sites for this analysis) and one site each in South Africa and Uganda (categorized as African region sites). All tuberculosis treatment was administered 5 days/week for 8 weeks and directly observed. All participants underwent HIV testing. Sputum cultures were done biweekly on both solid and liquid media during the first eight weeks of treatment. Overall, 92% of liquid cultures were performed in a BACTEC MGIT 960 system (Becton, Dickinson and Co., Franklin Lakes, NJ) or a BACTEC 460TB system (Becton, Dickinson and Co.). Types of solid medium used by local laboratories included Lowenstein-Jensen, Middlebrook 7H10, and Middlebrook 7H11. *M. tuberculosis* isolates underwent confirmatory drug-susceptibility testing at the Centers for Disease Control and Prevention (CDC) using the indirect agar proportion method for isoniazid, rifampin, ethambutol, and fluoroquinolones (ofloxacin and ciprofloxacin critical concentrations of 2µg/ml); MGIT 960 medium was used for pyrazinamide susceptibility testing. In Study 28, no significant difference was found in the rate of bacteriologic culture conversion after 8 weeks of treatment between patients taking either the moxifloxacin or isoniazid containing drug regimens [12]. However, patients enrolled at African sites were significantly more likely to remain sputum culture positive after 8 weeks of treatment than patients enrolled at non-African sites, even after adjustment for pulmonary cavitation, HIV infection, and history of treatment in the days prior to study enrollment.

### Selection of participants and Isolates

As in the primary analysis of Study 28, we included patient data and *M. tuberculosis* isolates from all 328 protocol correct participants (as defined by adherence to study treatment and report of all biweekly culture results) in the current study.

### Genotyping techniques

**Figure 1:**
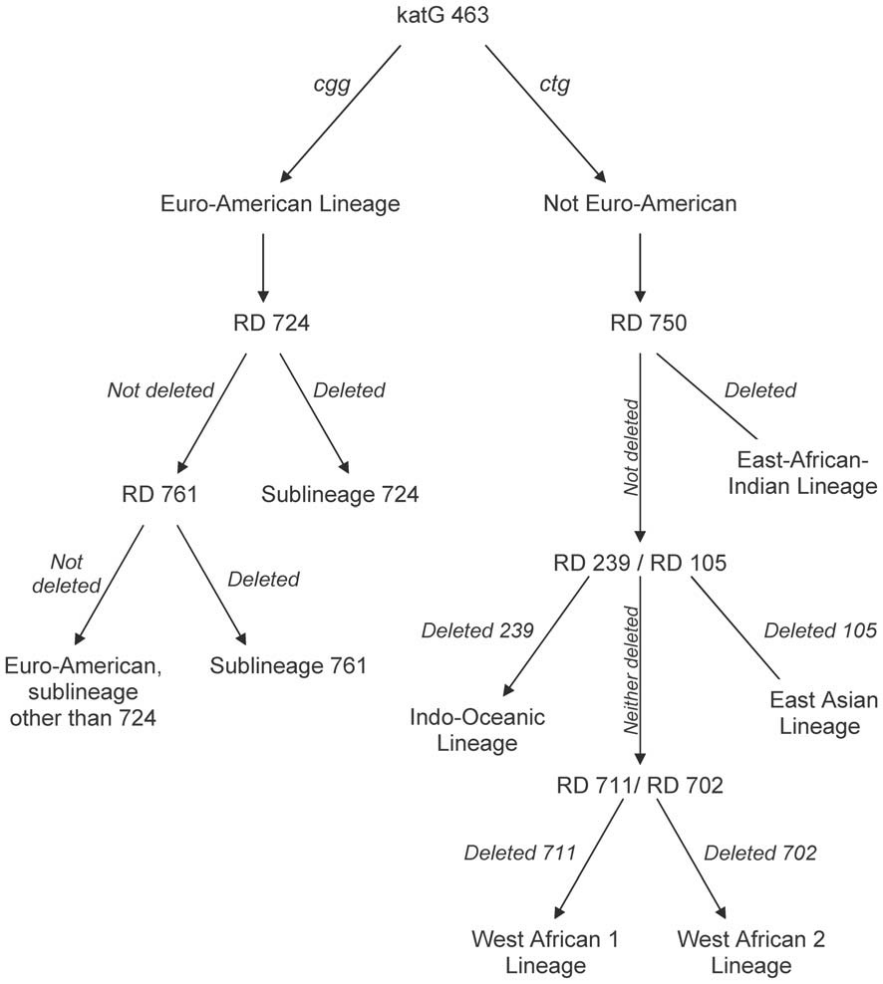
Algorithm applied to all *M. tuberculosis* isolates for the determination of lineage and sublineage.

Large sequence polymorphism (LSP) and single nucleotide polymorphism-based typing for the determination of the lineage and sublineage were performed as previously described [7], [13]. The MTB Research Laboratory at the University of California, San Francisco performed all genotyping and was blinded to the study site from which the isolates originated. Consequently, a systematic algorithm was applied to all isolates received from the CDC (Figure 1). Briefly, the katG 463 codon was analyzed using real time PCR. If the codon was defined as cgg [14] the strain was considered to belong to the Euro-American lineage. Because the Euro-American sublineage 724 has been described as predominant in Uganda [15] and Euro-American sublineage 761 predominant in South Africa [7], we next screened for these two sublineages. If the katG 463 codon was defined as ctg, we proceeded to screen for LSPs that define the East-African-Indian, Indo-Oceanic, East Asian, West African 1 and West African 2 lineages. PCR and/or multiplex real-time PCR (Applied Biosystems, Foster City, CA and Stratagene, La Jolla CA, USA) were used to determine the presence or absence of the RDs, using primers previously published [13].

### Statistical Analysis

The number of participants and isolates included was determined from the parent clinical trial. Race/ethnicity was determined by site staff using categories defined in the United States tuberculosis surveillance system. The definitions and primary objectives of this study were formulated prior to data analysis. All statistical analysis was done using SAS v.9.2 (Cary, NC). The initial analysis included describing the observed genotypes and their clinical presentation, stratified by region of enrollment (Africa versus non-Africa). Analysis of deviance (ANODEV), and Pearson's chi-square or Fisher's exact tests for differences among the lineages were used, as appropriate. Subsequent analyses were directed at evaluating the influence of *M. tuberculosis* lineage on treatment response as measured by 8-week culture status. Associations between lineage and 8-week culture conversion were evaluated using univariate logistic regression analysis, excluding West African lineages due to small numbers. Univariate analyses stratified by region of enrollment and presence of baseline cavitation were done to address potential confounding. Associations with 8-week culture conversion were adjusted in multivariate logistic regression models for factors found to be significant in the parent clinical trial.

## Results

### Distribution of lineages of *M. tuberculosis*

*M. tuberculosis* lineage/sublineage was successfully determined in 306 (93%) of 328 protocol correct participants enrolled in Study 28, with 192 (63%) enrolled at African sites [168 (88%) from Kampala, Uganda and 24 (12%) from Durban, South Africa] and 114 (37%) enrolled at Non-African sites. Genotype information was not available in 22/328 (9.2%) participants either because repeated attempts to re-grow the isolates were unsuccessful (n = 20) or the genotyping suggested mixed infection with more than one lineage of *M. tuberculosis* (n = 2). Of the 306 isolates successfully genotyped, 246 (80%) belonged to the Euro-American lineage, 101 of which were sublineage 724, and 145 isolates were Euro-American other than sublineage 724 (which included 3 isolates genotyped as Euro-American sublineage 761). Other lineages observed in this cohort included East-African-Indian (26, 8.5%), East Asian (22, 7.2%), Indo-Oceanic (11, 3.6%), and West African 1 (1 0.3%) and their specific geographic distribution is shown in Table 1.

**Table 1:** Distribution of lineages and sublineages of *M. tuberculosis* isolates obtained from TBTC Study 28 by region and country of enrollment.

*M. tuberculosis* lineage	All Study Participants	African RegionParticipants	Kampala, Uganda	Durban, South Africa	Non-African RegionParticipants	North America	Rio de Janeiro, Brazil	Barcelona, Spain
	n = 306	n = 192	n = 168	n = 24	n = 114	n = 83	n = 20	n = 11
**Euro-American - other than 724**	**145 (47%)**	**56 (29%)**	43 (26%)	13 (54%)	**89 (78%)**	58 (70%)	20 (100%)	11 (100%)
**Euro-American – 724**	**101 (33%)**	**99 (52%)**	99 (59%)	—	**2 (1.8%)**	2 (2.4%)	—	—
**East-African-Indian**	**26 (8.5%)**	**24 (12%)**	24 (14%)	—	**2 (1.8%)**	2 (2.4%)	—	—
**East Asian**	**22 (7.2%)**	**12 (6.2%)**	1 (0.6%)	11 (46%)	**10 (8.8%)**	10 (12%)	—	—
**Indo-Oceanic**	**11 (3.6%)**	**1 (0.5%)**	1 (0.6%)	—	**10 (8.8%)**	10 (12%)	—	—
**West African 1**	**1 (0.3%)**	**—**	—	—	**1 (0.9%)**	1 (1.2%)	—	—

The distribution of *M. tuberculosis* lineages and sublineages varied by geographic region. Euro-American sublineage 724 predominated among African region participants (99/192, 52%), whereas Euro-American other than 724 predominated among non-African region participants (89/114, 78%). The majority of Euro-American 724 (99/101, 98%) and East-African-Indian (24/26, 92%) lineage isolates were identified in African region participants. In contrast, the majority of Indo-Oceanic (10/11, 91%), Euro-American other than 724 (89/145, 61%), and West African 1 (1/1, 100%) were identified in non-African region participants (Table 1). East Asian lineage was found in equal frequency by region of enrollment, but in Africa, it was isolated primarily from participants enrolled in Durban, South Africa.

### Lineages of *M. tuberculosis* and presentation of disease

Disease characteristics including cavitation on baseline chest radiograph, extensive lung involvement, high bacillary load on baseline sputum smear (defined as 3+/4+ scoring of acid-fast bacilli in the smear), and presence of cough or fever at time of presentation were analyzed to determine if any association existed with lineage of *M. tuberculosis*. Overall, bacillary load on baseline sputum smear varied significantly by *M. tuberculosis* lineage (p = 0.01), but no significant associations between symptoms or radiographic findings and lineage were noted. When compared with patients with disease caused by Euro-American sublineages other than 724, patients with disease caused by the Indo-Oceanic lineage were less likely to present with high bacillary load on baseline sputum smear (OR 0.23, 95% CI 0.06–0.91, p = 0.03) while patients with disease caused by Euro-American sublineage 724 were more likely to present with high bacillary load (OR 1.76, 95% CI 1.01–3.08, p = 0.05). After stratifying by region, however, the overall association was no longer statistically significant (p = 0.09).

### Factors associated with positive cultures at week 8

In univariate analysis, two *M. tuberculosis* lineages were found to be associated with bacteriologic non-conversion at week 8 (p<0.01). Specifically, both Euro-American sublineage 724 (OR 2.38, 95% CI 1.41–4.01, p<0.01) and East Asian lineage (OR 3.11, 95% CI 1.24–7.79, p = 0.02) were significantly associated with a higher probability of positive week-8 culture when compared with Euro-American sublineages other than 724 (Table 2). However, adjustment for the risk factors for week-8 culture positivity from the parent study (African region of enrollment, cavitary disease on initial chest radiograph, high bacillary load on baseline sputum smear and age) resulted in lineages no longer being a significant risk factor for bacteriologic non-conversion at 8 weeks. Recognizing that lineage may lead to certain tuberculosis disease characteristics, we also performed this analysis excluding cavitation and bacillary load as covariates, and again found no association between lineage and week-8 culture positivity, after adjustment for region of enrollment and age.

**Table 2:** The association between week-8 culture positivity and *M. tuberculosis* lineage, adjusted for clinical risk factors from the parent study.

Characteristic	Positive week-8 culture[n (row %)]	Univariate Analysis		Multivariate Analysis	
		p-value	OR (95% CI)	p-value	OR (95% CI)
Moxifloxacin (n = 152)	56 (37)	0.19	0.74 (0.47–1.17)	0.23	0.73 (0.43–1.22)
**Assignment stratum**					
Non-African/non-cavitary (n = 32)	2 (6.3)		1.00 (ref)		1.00 (ref)
Non-African/cavitary (n = 82)	27 (33)	<0.01	7.36 (1.64–33.1)	0.02	5.93 (1.25–28.1)
African/non-cavitary (n = 47)	21 (45)	<0.01	12.1 (2.59–56.6)	0.01	7.61 (1.43–40.5)
African/cavitary (n = 145)	74 (51)	<0.01	15.6 (3.60–67.8)	<0.01	9.86 (1.99–48.9)
Age at enrollment (per year) *	33 (12)	0.95	1.00 (0.98–1.02)	0.06	1.03 (1.00–1.06)
High bacillary burden on baseline sputum smear (n = 205) *	100 (49)	<0.01	3.06 (1.79–5.21)	0.03	2.02 (1.09–3.76)
Days to detection in liquid culture system for baseline specimen *	7.1 (4.8)	<0.01	0.92 (0.87–0.96)	0.02	0.94 (0.90–0.99)
**Lineage**					
Euro-American – other than 724 (n = 145)	46 (32)		1.00 (ref)		1.00 (ref)
Euro-American – 724 (n = 101)	53 (52)	<0.01	2.38 (1.41–4.01)	0.12	1.70 (0.88–3.30)
East-African-Indian (n = 26)	11 (42)	0.29	1.58 (0.67–3.70)	0.91	0.95 (0.35–2.54)
East Asian (n = 22)	13 (59)	0.02	3.11 (1.24–7.79)	0.30	1.79 (0.60–5.37)
Indo-Oceanic (n = 11)	1 (9.1)	0.15	0.22 (0.03–1.73)	0.27	0.29 (0.03–2.58)

*Mean and standard deviation reported for continuous data.

Univariate analyses stratified by region of enrollment and presence of cavitation on baseline chest radiograph were conducted to identify the contribution of lineage for stratum-specific differences in week-8 culture status (data not shown). Overall, lineage was not associated with increased likelihood of positive week 8 culture within any of the region of enrollment/presence of cavitation strata. However, the data suggest (with a wide confidence interval) that among patients enrolled from African sites who had cavitary tuberculosis, disease caused by East Asian lineage was associated with increased odds of positive week 8 culture when compared to persons with disease caused by Euro-American sublineages other than 724 (OR 10.7, 95% CI 1.22–93.1).

## Discussion

In this study, our primary objective was to describe the lineages and sublineages of isolates of *M. tuberculosis* from participants in an international, randomized, tuberculosis treatment trial. Our secondary objectives included exploring differences in disease presentation by lineage, and determining whether lineage of the infecting strain of *M. tuberculosis* is associated with the likelihood of bacteriologic culture conversion at 8 weeks as a marker for response to treatment. Examining isolates from Study 28 provided an opportunity to address this question in tuberculosis patients who were well-characterized and in whom treatment and follow-up was assured in a systematic manner, thus addressing potential confounders of treatment response.

For our first objective, we found that the most common genotype was the Euro-American lineage, comprising 80% of isolates in Study 28. The geographic distribution of sublineages within the Euro-American lineage and that of other lineages was unequal. Euro-American sublineage 724 was found primarily in study participants enrolled in Kampala, Uganda. This finding is in line with a recent report in which Euro-American sublineage 724 was designated the “Uganda genotype”, due to its high prevalence in patients with tuberculosis in that country, which included, among others, what was previously known as spoligotype *M. africanum* subtype Uganda I and II [15]. In our study, two other lineages identified with near exclusivity to a site or region: 92% (24/26) of the isolates belonging to the East-African-Indian lineage were identified in participants enrolled in Kampala, Uganda, and 91% (10/11) of the isolates belonging to the Indo-Oceanic lineage were identified in participants enrolled in North American sites. Isolates belonging to the East Asian lineage, which includes the Beijing spoligotype family [16], [17], were found in equal frequency by region of enrollment, but in Africa, isolates from this lineage were mainly identified from participants enrolled in South Africa, where the East Asian lineage was recently identified as an emerging genotype [18]. The causes for such lineage-specific associations with geographic regions are unknown. It has been suggested that the biogeography of *M. tuberculosis* strains are linked to ancient human migrations [1], and that strains may have adapted to specific human host populations over time [7]. The causes not withstanding, it is important to recognize that within the context of a clinical trial conducted across diverse geographic locations, genotypes of *M. tuberculosis* varied and that some lineages and sublineages were exclusive to specific sites.

Our secondary objectives for this study were to explore the impact of lineage on presentation of disease and response to treatment in Study 28. We found that the lineage of *M. tuberculosis* did not independently influence presentation of disease, nor response to treatment as measured by week-8 culture status as the primary endpoint. However, we believe our study is underpowered to definitively assess the relationship between lineage and treatment response as genotyping information identified a co-linear relationship between site of enrollment and lineage/sublineage. In stratified univariate analyses we found that among patients with cavitary tuberculosis enrolled from African sites, persons with disease caused by East Asian lineage were more likely to have a positive week-8 culture when compared to persons with disease caused by Euro-American sublineages other than 724. This finding, though exploratory in nature and not statistically significant, is in line with other recent studies that identified clinical and epidemiologic consequences of specific host-pathogen relationships in tuberculosis [7], [19], [20], [21], [22]. Gagneux and colleagues, recently showed that lineages of *M. tuberculosis* varied in regard to pathogenicity (defined as the ability of *M. tuberculosis* to cause secondary cases), and that this variation was driven in part by specific relationships between the ethnicity of the host and the phylogeographic grouping of *M. tuberculosis* [7]. Thwaites et al [20], identified an association between the East Asian lineage and disease progression in tuberculosis meningitis, while Caws et al [19], found that tuberculosis caused by isolates belonging to the Euro-American lineage was limited and protective against disseminated, meningeal tuberculosis in Vietnamese adults. Burman et al, performed an evaluation of the role of host-pathogen relationships in regard to risk for relapse in a phase 3 treatment trial and found that patients with tuberculosis caused by strains from the Beijing family (a member of the East Asian lineage) had a higher risk for relapse, even after adjustment for factors previously associated with relapse. Interestingly, the association between lineage and relapse was found to be even stronger when strains from the Beijing family caused disease among Asian–Pacific Islanders [21]. Finally, our data suggesting that disease caused by East Asian lineage isolates in cavitary, African-region participants may be less responsive to combination therapy is in line with another recent report from South Africa that found patients with disease caused by members of the Beijing family had a lower likelihood of being successfully treated [22].

Our study has several limitations. First, the hypotheses for the study were proposed post-hoc. We took advantage of the fact that *M. tuberculosis* isolates had been collected and stored from well-characterized participants in a clinical trial; however, the trial itself was not designed to test the proposed hypotheses, and our study was limited by the sample size of Study 28. Second, our primary endpoint was 2 month culture status, not failure or relapse. In the study by Burman et al [21], strains belonging to the East Asian lineage were associated with increased risk of relapse and failure, but not with delayed conversion at week-8 cultures. Study 28 was a phase 2 trial and did not follow patients through to relapse. Third, whereas our clinical, microbiologic and radiographic data were robust as part of the routine clinical trial data collection process, we did not have measures of nutritional status or co-existing parasitic infections that might have influenced severity of disease at presentation and potentially response to treatment. Fourth, although there is some evidence that genotype may play a role in pathogenicity, we do not have tools to confirm that all strains belonging to a particular lineage will have the same pathogenicity factors.

In summary, we have shown that within the context of a clinical trial conducted across diverse geographic locations, genotypes of *M. tuberculosis* varied and some lineages and sublineages were exclusive to specific trial sites. We found interesting lineage-associated variations in disease presentation and week-8 culture positivity that were not statistically significant when adjusted for clinical factors and region of enrollment. This highlights the importance of examining for other factors that potentially impact treatment outcomes when evaluating the relationship between genotype and response to tuberculosis therapy. Additional studies are needed to definitively determine whether phylogenetic lineage influences treatment outcomes including failure and risk for relapse.
